# Infections in Pediatric Palliative Care Units: Clinical and Microbiological Perspectives from a Single Center

**DOI:** 10.3390/antibiotics15030261

**Published:** 2026-03-03

**Authors:** Sefika Aldas, Merve Türkegün Şengül, Berfin Ozgökçe Ozmen, Sanlıay Sahin

**Affiliations:** 1 Department of Pediatrics, University of Health Sciences, Mersin City Education and Research Hospital, 33800 Mersin, Turkey; dr.b.ozmen@hotmail.com (B.O.O.); sanliay@yahoo.com (S.S.); 2 Department of Biostatistics and Medical Informatics, Faculty of Medicine, Alaaddin Keykubat University, 07425 Alanya, Turkey; merveturkegun@gmail.com

**Keywords:** care, child, infection, multidrug resistance, palliative, pediatrics

## Abstract

**Aim**: Infections and multidrug-resistant (MDR) pathogens are concerns in pediatric palliative care (PPC) units, where children with life-limiting conditions undergo invasive procedures and prolonged hospitalization. This study evaluated clinical characteristics, microbiological profiles, and factors associated with MDR infections among pediatric patients hospitalized in a PPC unit. **Methods**: This retrospective observational study included 66 children aged 1 month to 18 years who were admitted to the PPC unit of our hospital due to infection between June 2023 and January 2024. Demographic data, comorbidities, device use, infection sites, and microbiological results were reviewed. Bacterial identification and antimicrobial susceptibility testing were performed using the Vitek2 system and interpreted according to EUCAST. **Results**: The median age was 48 months (IQR 19–106); 63.6% were male. Lower respiratory tract infection was most common (68.2%), followed by sepsis (13.6%) and urinary tract infection (12.1%). *Pseudomonas aeruginosa* (36.4%) and *Klebsiella pneumoniae* (27.3%) predominated. MDR organisms represented 15.2% of isolates. MDR infections were significantly associated with percutaneous endoscopic gastrostomy or mechanical ventilation use (*p* = 0.033). Prolonged hospitalization and multiple comorbidities tended to increase the MDR risk but did not reach statistical significance. **Conclusions**: Gram-negative MDR infections constitute an important problem in PPC units. Frequent exposure to invasive devices and antibiotics increases susceptibility to resistant pathogens. Reinforcing infection prevention, optimizing antimicrobial stewardship, and monitoring device-related infections are essential to reduce morbidity and improve care quality in pediatric palliative care.

## 1. Introduction

The World Health Organization (WHO) defines pediatric palliative care (PPC) as a multidisciplinary healthcare service that aims to improve the quality of life of children and their families, which focuses on the physical, psychological, and psychosocial needs of children struggling with life-limiting and chronic diseases [1]. Thanks to advances in technology, many children require multiple treatments in PPC due to serious and life-threatening chronic conditions such as cancer, congenital/genetic disorders, and neuromuscular diseases [2,3]. PPC units aim to manage the symptoms experienced by children, control pain, and provide psychosocial support. Among the life-threatening health problems for children with chronic diseases, infections constitute a significant problem in PPC units [4]. Factors such as frequent and prolonged hospitalization, multiple surgical interventions, exposure to intensive antibiotic treatment and immunosuppressive therapies, and the use of long-term medical devices such as nasogastric (NG) catheters, percutaneous endoscopic gastrostomy (PEG) tubes, central venous catheters (CVCs), tracheal cannulas, and ventriculoperitoneal (VP) shunts increase the risk of colonization by microorganisms showing multidrug resistance (MDR). The emergence of multidrug-resistant organisms (MDROs) is a major public health concern [5]. The presence of resistance to one agent from at least 3 antimicrobial categories is defined as MDR [6]. Pathogens showing MDR do not respond to standard antibiotic treatments and complicate treatment processes [7,8]. According to international CDC/EUCAST definitions, multidrug-resistant (MDR) microorganisms are non-susceptible to at least one agent in three or more antimicrobial categories. Accordingly, pathogens such as methicillin-resistant *Staphylococcus aureus* (MRSA) and vancomycin-resistant enterococci (VRE) are classified as MDR due to resistance to key antimicrobial agents [9,10].

MDR infections have clinical symptoms similar to diseases caused by susceptible pathogens, and treatment options for these infections are extremely limited. In recent years, there has been a marked increase in the prevalence of antibiotic-resistant bacteria in healthcare settings. The proportion of extended-spectrum β-lactamase (ESBL)-producing *Escherichia coli* and *Klebsiella pneumoniae* strains was reported to be 42.5% and 30.2%, respectively. Carbapenem-resistant *Enterobacterales* (CRE) infections pose a serious threat, especially in Europe. Furthermore, the prevalence of Gram-positive pathogens such as methicillin-resistant *Staphylococcus aureus* (MRSA) and vancomycin-resistant *Enterococcus* spp. (VRE) is also increasing. Among non-fermenting Gram-negative bacteria, the prevalence of multidrug-resistant *Pseudomonas aeruginosa* and *Acinetobacter baumannii* strains has also reached alarming levels [11,12,13,14,15]. Therefore, this study aimed to determine the prevalence of infections in PPC units and to analyze the characteristics of these infections comprehensively. In this context, the demographic characteristics of pediatric patients with life-threatening diseases, the frequency of infections during hospitalization, and the distribution of microorganisms isolated are evaluated to increase the life expectancy of these vulnerable children.

## 2. Results

Sixty-six patients who met the study criteria and who were hospitalized in the PPC ward for six months were included in the study. The cohort was predominantly male, with a wide age distribution, and more than half of the patients had multiple comorbid conditions. The median age of girls was 54 (32.3–132) months; that of boys was 43 (19.3–94) months, and analysis of the median ages was not statistically significant (*p* = 0.476). Lower respiratory tract infections were the most frequent admission diagnosis. Multidrug-resistant (MDR) organisms were detected in 10 patients. Among these patients, infections were most frequently caused by *Klebsiella pneumoniae* and *Pseudomonas aeruginosa*, with urine and blood being the most common sites of isolation. Demographic and clinical characteristics of all patients with MDR infections are summarized in Table 1.

**Figure 1:**
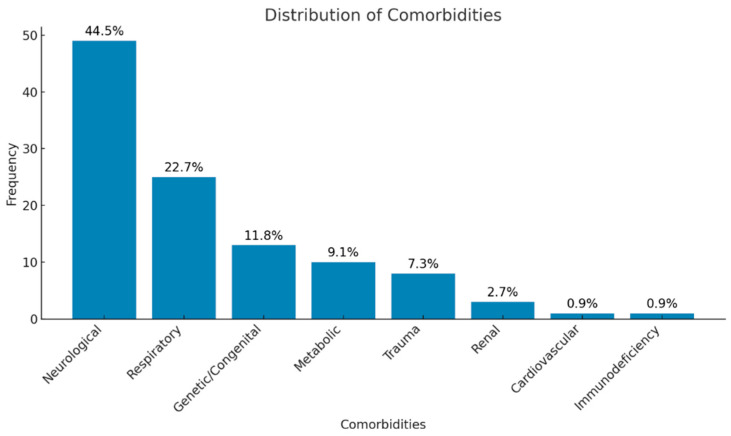
Distribution of comorbidities among all patients.

Comorbidities were analyzed using a multiple-response approach, allowing more than one diagnosis per patient. A total of 110 comorbid diagnoses were recorded in 66 children, classified into eight palliative condition categories. The neurological disorders were the most frequent underlying condition, followed by respiratory and congenital disorders. The overall distribution of comorbidities differed significantly across categories (*p* < 0.001). The distribution of comorbid diagnoses is illustrated in Figure 1.

Clinical evaluation at admission showed that fever, cough, and increased respiratory secretions were the most common findings. A history of recent hospitalization and prior antibiotic use was also frequently observed. A summary of clinical findings and positive physical examination results for all patients with MDR infections is provided in Table 2.

Most patients received single-agent antibiotic therapy, while a smaller proportion required combination treatment. Meropenem was the most frequently used antibiotic. Multidrug-resistant pathogens were identified in a limited subset of patients. The frequency of antibiotic use and MDR prevalence are shown in Table 2.

Laboratory analyses commonly demonstrated elevated inflammatory markers, particularly C-reactive protein. Most hematological and biochemical parameters were within or near reference ranges. Laboratory findings of the study cohort are summarized in Table 3.

The use of invasive medical devices was common. Statistically significant associations were observed between PEG use and ventilator dependence and between the distribution of culture sites among all patients and tracheal aspirate and blood samples. No statistically significant associations were identified for other medical devices. The association between medical device type and culture site distribution for all patients is presented in Table 4.

In patients with MDR infections, antibiotic use patterns and device-associated culture results were further analyzed. The distributions of device use and microbial isolation sites are shown in Table 4.

The distribution of bacterial species isolated from different medical devices among all patients and those with multidrug-resistant (MDR) isolates is summarized in Table 5. The most frequently isolated pathogens varied according to the type of medical device used. Among patients with MDR isolates, Gram-negative bacteria constituted the majority of the isolates, with notable differences in species distribution compared to the overall patient population.

The distribution of bacterial species isolated from different medical devices did not differ significantly (*p* > 0.05).

The antibiotic resistance profiles of the isolated microorganisms, evaluated using multiple response analysis, are presented in Table 6. Resistance was observed against several antibiotic classes, reflecting the multidrug-resistant patterns among the isolates.

Firth’s penalized logistic regression analysis did not identify statistically significant associations between the number of comorbidities or the duration of hospitalization and the occurrence of MDR infections. Although longer hospital stays and a higher number of comorbidities were associated with higher odds of MDR infection, these associations did not reach statistical significance (Table 7).

## 3. Discussion

Treatment processes in PPC include a multidisciplinary approach aiming to apply various medical devices supporting vital functions to patients, improvement with medical treatments in disease processes requiring hospitalization, treatment of infections, pain control, and improvement of the patient’s quality of life [16]. This study aimed to evaluate the infections observed in patients with life-threatening diseases who were monitored in the PPC unit because of infectious diseases.

In our study, male patients represented a larger proportion of the cohort, indicating a male predominance compared to female patients. Similar gender distributions have been reported in previous multicenter studies of PPC populations [17]. Although it is thought that these differences may be related to biological or sociocultural factors or inequalities in access to health services, future research is needed in this regard.

In the study, it was observed that patients hospitalized in the PPC unit mostly had neuromuscular diseases. This was followed by respiratory diseases and genetic/metabolic diseases. The predominance of neuromuscular diseases in this patient group is a finding frequently emphasized in the literature. Similarly, neuromuscular and cardiovascular diseases are among the most common diagnoses in PPD centers in different studies [18]. This may be related to the high morbidity rates and long-term care requirements of neuromuscular diseases. Complications such as loss of motor function, feeding difficulties, and respiratory failure, which are frequently encountered in this disease group, increase the need for palliative care. Our results are important in terms of the predictability of the need for palliative care in this patient group, and we think that these findings should be considered in the planning and management of palliative care services.

The infectious diagnoses of the patients were determined by considering clinical features, laboratory results, and culture findings, and it was found that the most common infections were LRTI and sepsis. According to the literature, factors such as thoracic and spinal deformities, accumulation of secretions, and recurrent aspirations increase the risk of developing lower respiratory tract infections in pediatric palliative care patients [19,20,21]. The increased frequency of infection, especially among ventilator-dependent patients with tracheostomies, may be associated with the fact that these patients are constantly monitored with invasive devices and have weak immune systems.

The high rate of CVC use in our study was compatible with the literature [22]. The need to continue treatments such as intravenous sedation, analgesia, and infusion of antiepileptic drugs in patients stabilized in the PICU and transferred to the PPC unit may have increased the use of CVC. This situation shows that, after intensive care, PPC patients continue to need advanced treatment, and these processes should be carefully managed.

In our study, the rates of tracheostomy and mechanical ventilation support were found to be consistent with similar studies in the literature [3,23,24]. In a PPC study conducted in 2023, it was reported that a significant proportion of patients needed respiratory support, and this support was provided by invasive and non-invasive methods [25]. In our study, it was found that patients mostly presented with respiratory complaints, and especially increased secretion, cough, and fever were the most common symptoms. Similarly, in the literature, it has been reported that respiratory problems and fever were the most common symptoms in PPC patients [26]. This might be due to an inadequacy in secretion clearance and cough reflexes in the patients.

When the absolute lymphocyte and neutrophil counts of our patients were evaluated, it was observed that almost half of them were within normal limits. However, thrombocytopenia and thrombocytosis were also observed. In the literature, the prevalence of thrombocytopenia has been particularly observed in septic shock patients [27]. The fact that sepsis may cause a decrease in platelet count by affecting the hematological system has been supported by the significant relationship between septic patients and low platelet levels in some studies [3,23,24,28]. This finding is important in terms of clinical evaluation. In our patients, low hemoglobin and hematocrit values were observed only in a small group. According to the literature, this is thought to be related to anemia developing due to underlying chronic diseases [29]. In addition, CRP elevation was found in the majority of our patients. It is emphasized in the literature that this is important in supporting the diagnosis and predicting the prognosis in cases of sepsis and septic shock [30,31]. The presence of elevated CRP is a red flag regarding severe bacterial infection. In addition, elevated liver and renal function tests can be observed in some of the patients, so these data should be monitored carefully for clinical prognosis.

In the cultures obtained from the patients in our study, positive results were most frequently detected in blood, tracheal aspirate, urine, and CVC samples. The fact that a significant portion of culture positivity in our study was obtained from tracheal aspirate and bronchoalveolar lavage samples highlights the difficulty in distinguishing between infection and colonization, particularly in patients with tracheostomy and mechanical ventilation support. To make this distinction, microbiological findings, clinical symptoms, inflammatory markers, and radiological findings must be evaluated together. However, it is known that airway colonization cannot be completely ruled out in children who are chronically ventilator-dependent [32]. *Pseudomonas aeruginosa* and *Klebsiella pneumoniae* were the predominant microorganisms, while *Acinetobacter baumannii*, *Stenotrophomonas maltophilia*, and *Escherichia coli* were isolated less frequently. In light of these findings, it is recommended that blood and urine cultures should be taken during the hospitalization of all patients.

In this study, empirical antibiotic treatment was initiated in all patients upon hospitalization.

In this study, multidrug resistance was assessed based on internationally accepted consensus definitions. Accordingly, MDR microorganisms were defined as isolates that are non-susceptible to at least one agent in three or more antimicrobial categories. Intermediate susceptibility results were also considered non-susceptible and evaluated within the MDR definition. According to the antimicrobial susceptibility test lists for microorganisms established by the U.S. Food and Drug Administration (FDA), the European Committee on Antimicrobial Susceptibility Testing (EUCAST), and the Clinical and Laboratory Standards Institute (CLSI), MDR is defined as resistance to at least one agent in ≥3 antimicrobial categories [10]. MDR-GNBs are liable for more than 50% of healthcare-associated infections and induce mortality rates ranging from 30% to 70% [33]. Resistance to most classes of antibiotics results in limited therapy options and has led to the reevaluation and reuse of colistin, which is effective against most MDR-GNB [34]. 

In our study, colistin therapy was found to be administered to 50% of MDR patients. In general, broad-spectrum antibiotics, particularly meropenem and colistin, were frequently used in the study population. However, due to the retrospective nature of the study and the treatment duration, a detailed antimicrobial stewardship analysis could not be performed due to the lack of standardized data on de-escalation practices and the appropriateness of empirical treatment. Nevertheless, treatment based on culture results in PPC patients, regular reassessment of empirical regimens, and implementation of de-escalation strategies are considered critical in reducing exposure to broad-spectrum antibiotics. Prospective studies in this area will significantly contribute to optimizing antimicrobial use in PPC units. The most common sites of isolation of MDR microorganisms were blood and urine cultures. Similar results of MDR microorganisms in PPC patients have been reported in the literature [4,35]. Frequent isolation of MDR pathogens, especially *Pseudomonas aeruginosa* and *Klebsiella pneumoniae* species, in PPC units causes serious difficulties in antibiotic treatment. Carbapenem resistance makes the treatment of these pathogens more complex and leads to limited alternative treatment options. It is also reported in the literature that MDR pathogens are isolated more frequently, especially in palliative care patients, and this situation makes infection management difficult. In addition, the use of medical devices stands out as one of the most important factors that increase the risk of colonization or infection with MDR pathogens in these patients [4,36,37]. More studies are required in this field, since MDRs have been associated with increased hospitalization times, costs, and mortality [38]. In a New York-based intensive care study, it was documented that MDR Gram-negative bacilli were associated with increased mortality, length of hospitalization, and hospital costs [39].

In our study, patients with multidrug-resistant infections accounted for 16.6% of the total cohort. On detailed evaluation, we found that mortality rates and length of hospitalization in MDR patients were inconsistent with the available literature [40,41]. This discrepancy may be attributed to the retrospective nature of the study, possible data limitations in the medical records, and the relatively small number of cases of MDR included in our cohort.

This study was designed with a primarily exploratory and descriptive aim, intended to characterize clinical patterns and potential associations rather than to establish definitive or causal relationships. The findings provide an overview of the frequency of infections observed in PPC units, highlighting the presence of multidrug-resistant pathogens in this setting. Resistant organisms may pose challenges for infection management, so it is important to emphasize the development of more effective strategies for infection control in these units. Future research with larger samples is needed with multicenter cohorts to improve the effectiveness of infection control measures.

In conclusion, the prevention of infections in PPC units should be addressed continuously with a multidisciplinary approach. In this context, it is vital to update infection control policies, strictly supervise hand hygiene practices, take appropriate isolation measures, and carefully monitor the use of invasive devices. The implementation of effective infection control strategies plays a critical role in preventing and combating diseases in children with special needs.

### Limitations

This study has several limitations, including its retrospective, single-center design and relatively small sample size, which may limit its generalizability.

## 4. Methods and Materials

This study was conducted on 66 patients who were treated for infection among a total of 158 patients aged between 1 month and 18 years, who were followed up for six months between 1 June 2023 and 1 January 2024 in the Pediatric Palliative Care inpatient clinic of Mersin City Training and Research Hospital. After the approval of the Local University Ethics Committee (Protocol No: E-26.01.2024/7), the detailed file data of the patients hospitalized in the PPC inpatient clinic were retrospectively scanned from our hospital’s database. Patients with incomplete or inadequate data, newborns, patients over 18 years of age, and those with repeated hospitalizations were excluded from the study. For this purpose, a standardized data collection form was used to obtain demographic, clinical, and infection-related data from medical records. Within the scope of the study, demographic characteristics such as age, gender, diagnoses (metabolic, neurological, genetic, respiratory, gastrointestinal, cardiovascular, hematological/oncological, renal, endocrinological, and other causes such as trauma), reasons for hospitalization leading to infection, caregivers, cities of residence and nationalities, the medical/technological devices that they had to use and the number of these devices, the way of presentation of complaints, physical examination findings, laboratory test results, blood, urine, and stool culture results, secondary infections during hospitalization, and infection status with MDR were recorded in detail. For the inoculation of clinical samples, 5% sheep blood agar (RTA, Kocaeli, Turkey) and eosin methylene blue (RTA, Turkey) media were used. The cultured samples were incubated at 37 °C for 18–24 h and then evaluated by a specialist in microbiology. Vitek2 (BioMérieux, Craponne, France) automated systems were used for the identification of bacterial species and determination of antibiotic susceptibility of bacteria, and antibiotic susceptibilities were interpreted according to the European Committee on Antimicrobial Susceptibility Testing (EUCAST) criteria. All microbiological cultures, bacterial identification, and antimicrobial susceptibility testing were performed as part of routine clinical care, and no additional procedures were carried out for research purposes.

### Statistical Analysis

All data were analyzed using SPSS 28.0. The distribution of numerical variables was evaluated by the Shapiro–Wilk test. Numerical variables were summarized as median, first (Q1), and third quartile (Q3) values, and categorical variables as numbers and percentages. Multiple response analyses were performed considering antibiotic use and many comorbidities with more than one outcome, and bar graphs were drawn. Two independent groups were compared by the Mann–Whitney U test. The association between medical device type and culture site distribution was assessed only in the overall cohort (n = 66) using the chi-square test of homogeneity; no statistical comparisons were performed in patients with MDR (n = 10) because of the limited sample size. When the assumptions of the chi-square test were violated due to sparse cell counts while assessing the association between medical device type and bacterial species, the Fisher–Freeman–Halton exact test was applied in the overall cohort (n = 66; Table 5), whereas this test was not used in patients with MDR (n = 10) because of the limited sample size. Differences in proportions between two categories were assessed using the two-proportion Z test.

Possible risk factors for MDR were explored using Firth’s penalized logistic regression to reduce small-sample bias and address separation. Analyses were performed using the *logistf* package in R (version 4.3.2). Odds ratios (ORs) with 95% confidence intervals (CIs) were estimated. A *p* < 0.05 was considered statistically significant for all comparisons.

## Figures and Tables

**Table 1:** Demographic and clinical characteristics of patients hospitalized in the PPC inpatient clinic.

		All Patients (n = 66)	Patients with MDR (n = 10)
**Age (months) Median (Q1–Q3)**		52 (19.3–106)	40 (11.75–87.75)
**Hospitalization (day) Median (Q1–Q3)**		15 (9–21)	19 (9.25–28.5)
		**n (%)**	**n (%)**
**Gender**	Female	24 (36.4)	4 (40.0)
	Male	42 (63.6)	6 (60.0)
**Child’s Nationality**	Turkish	49 (74.2)	9 (90.0)
	Syrian	17 (25.8)	1 (10.0)
**Comorbidity**	One	29 (43.9)	4 (40.0)
	Multiple	37 (56.1)	6 (60.0)
**Diagnosis**	LRTI	48 (72.7)	4 (40.0)
	Sepsis	9 (13.6)	1 (10.0)
	UTI	8 (12.1)	5 (50.0)
	AGE	1 (1.5)	---
**NG**		14 (21.2)	2 (20.0)
**PEG**		26 (39.4)	1 (10.0)
**Tracheostomy**		29 (43.9)	4 (40.0)
**VP Shunt**		8 (12.1)	2 (20.0)
**CVC**		41 (62.1)	3 (30.0)
**Ventilator Dependent**		24 (36.4)	3 (30.0)
**NIV**		17 (25.8)	3 (30.0)
**Site of isolation**	Tracheal aspirate/BAL	34 (51.5)	2 (20.0)
	Blood	19 (28.8)	3 (30.0)
	Urine	13 (19.7)	5 (50.0)

LRTI: lower respiratory tract infection, UTI: urinary tract infection, AGE: acute gastroenteritis, NG: nasogastric feeding tube, PEG: percutaneous enteral gastrostomy, NIV: non-invasive ventilation, CVC: central venous catheter, VP Shunt: ventriculoperitoneal shunt, BAL: bronchoalveolar lavage.

**Table 2:** Evaluation of patients’ clinical characteristics, physical examination findings, and isolated microorganisms.

		All Patients (n = 66)	Patients with MDR (n = 10)
		**n (%)**	**n (%)**
**Fever**		55 (83.3)	9 (90.0)
**Runny nose**		7 (10.6)	1 (10.0)
**Cough**		50 (75.8)	3 (30.0)
**Increased secretion**		53 (80.3)	4 (40.0)
**Vomiting**		24 (36.4)	8 (80.0)
**Convulsion**		23 (34.8)	3 (30.0)
**Diarrhea**		3 (4.5)	---
**Vaccination**	Missing	27 (40.9)	4 (40.0)
	Unvaccinated	39 (59.1)	6 (60.0)
**Previous history of antibiotic use**		16 (24.2)	1 (10.0)
**History of the previous hospitalization**		26 (39.4)	4 (40.0)
**Cutis marmorata**		23 (34.8)	4 (40.0)
**Tachycardia**		51 (77.3)	8 (80.0)
**Antibiotic Treatment**	One	56 (84.8)	
	1>	10 (15.2)	
**MDR**		10 (15.2)	
**Isolated Microorganism**	*Pseudomonas aeruginosa*	24 (36.4)	2 (20.0)
	*Klebsiella pneumoniae*	18 (27.3)	5 (50.0)
	*Acinetobacter baumannii*	7 (10.6)	1 (10.0)
	*Stenotrophomonas maltophilia*	6 (9.1)	---
	*Escherichia coli*	6 (9.1)	2 (20.0)
	*Serratia marcescens*	2 (3.0)	---
	*VRE*	1 (1.5)	---
	*Enterobacter cloacae*	2 (3.0)	---

MDR: Multi-drug resistant, *VRE*: vancomycin-resistant *Enterococcus* spp.

**Table 3:** The distribution of laboratory results for all patients.

	Within Normal Limits n (%)	Above/Below Expected Levels n (%)
**Neutrophils**	59 (89.4)	7 (10.6)
**Lymphocytes**	54 (81.8)	12 (18.2)
**Platelets**	51 (77.3)	15 (22.7)
**Hemoglobin**	42 (63.6)	24 (36.4)
**Hematocrit**	42 (63.6)	24 (36.4)
**C-Reactive Protein**	2 (3.0)	64 (97.0)
**Liver Function Tests**	42 (63.6)	24 (36.4)
**Renal Function Tests**	61(92.4)	5 (7.6)
**White Blood Cell (×10** **^3^ μL)** **Median (Q1–Q3)**	13,380 (9240–19,365)	
	**Yes** **n (%)**	**No** **n (%)**
**Oxygen support**	56 (84.8)	10 (15.2)
**Nutrition**	64 (97.0)	2 (3.0)
**Hydration**	66 (100)	NA

**Table 4:** The relationship between medical device type and culture site distribution.

Site of Culture					Site of Culture		
All Patients (n = 66)					Patients with MDR (n = 10)		
Medical Device	Tracheal Aspirate/BAL n (%)	Urine n (%)	Blood n (%)	* *p*-Value	Tracheal Aspirate/BAL n (%)	Urine n (%)	Blood n (%)
NG	6 (17.6)	2 (15.4)	6 (33.3)	0.436	0 (0.0)	0 (0.0)	2 (16.7)
PEG	16 (47.1)	1 (7.7)	9 (47.4)	0.033	0 (0.0)	0 (0.0)	1 (8.3)
Tracheostomy	15 (44.1)	3 (23.1)	11 (57.9)	0.150	0 (0.0)	1 (12.5)	3 (25.0)
Ventilator	13 (38.2)	1(7.7)	10 (52.6)	0.033	0 (0.0)	0 (0.0)	3 (25.0)
NIV	12 (35.3)	1 (7.7)	4 (21.1)	0.103	2 (100.0)	1 (12.5)	0 (0.0)
VP Shunt	3 (8.8)	4 (30.8)	1 (5.3)	0.102	0 (0.0)	2 (25.0)	0 (0.0)
CVC	20 (58.8)	7 (53.8)	14 (73.7)	0.446	0 (0.0)	4 (50.0)	3 (25.0)

CVC: central venous catheter, NIV: non-invasive ventilator, NG: nasogastric feeding tube, VP Shunt: ventriculoperitoneal shunt, PEG: percutaneous enteral gastrostomy, BAL: bronchoalveolar lavage. * *p*-values were obtained using the chi-square test to evaluate the association between medical device type and culture site in the overall patient cohort (n = 66). No statistical comparisons were performed in patients with MDR (n = 10) because of the limited sample size.

**Table 5:** Distribution of bacterial species isolated from different medical devices among all patients and patients with MDR isolates.

		*Pseudomonas aeruginosa* n (%)	*Klebsiella pneumoniae* n (%)	*Acinetobacter baumannii* n (%)	*Stenotrophomonas maltophilia* n (%)	*Escherichia coli* n (%)	*Serratia marcescens* n (%)	*VRE* n (%)	*Enterobacter cloacae* n (%)	* *p*
**All patients** **n = 66**	NG	2 (8.3)	6 (33.3)	2 (28.6)	3 (50.0)	0 (0.0)	0 (0.0)	1 (100.0)	0 (0.0)	0.930
	PEG	13 (54.2)	5 (27.8)	3 (42.9)	1 (16.7)	3 (50.0)	1 (50.0)	0 (0.0)	0 (0.0)	
	Tracheostomy	14 (58.3)	7 (38.9)	2 (28.6)	1 (16.7)	3 (50.0)	1 (50.0)	0 (0.0)	1 (50.0)	
	Ventilator	13 (54.2)	4 (22.2)	2 (28.6)	1 (16.7)	2 (33.3)	1 (50.0)	0 (0.0)	1 (50.0)	
	NIV	5 (20.0)	6 (33.3)	2 (28.6)	2 (33.3)	0 (0.0)	0 (0.0)	1 (100.0)	1 (50.0)	
	VP Shunt	3 (12.5)	3 (16.7)	0 (0.0)	0 (0.0)	1 (16.7)	0 (0.0)	1 (100.0)	0 (0.0)	
	CVC	16 (66.7)	10 (55.6)	3 (42.9)	5 (83.3)	4 (66.7)	1 (50.0)	1 (100.0)	1 (50.0)	
**Patients with MDR** **n = 10**	NG	1 (20.0)	3 (100)	0 (0.0)	0 (0.0)					
	PEG	3 (60.0)	0 (0.0)	1 (100.0)		0 (0.0)				
	Tracheostomy	3 (60.0)	2 (66.7)	0 (0.0)		0 (0.0)				
	Ventilator	3 (60.0)	2 (66.7)	0 (0.0)		1 (100.0)				
	NIV	1 (20.0)	1 (33.3)	1 (100.0)		0 (0.0)				
	VP Shunt	1 (20.0)	0 (0.0)	0 (0.0)		0 (0.0)				
	CVC	5 (100.0)	2 (66.7)	1 (100.0)		1 (100.0)				

*VRE*: vancomycin-resistant *Enterococcus*. * *p*-value was obtained from Fisher–Freeman–Halton exact test in the overall cohort (n = 66); no statistical comparisons were performed in patients with MDR (n = 10) because of the limited sample size.

**Table 6:** Antibiotic resistance profiles of isolated microorganisms based on multiple response analysis.

	Antibiotics	*P. aeruginosa* n (%)	*K. pneumoniae* n (%)	*A. baumannii* n (%)	*S. maltophilia* n (%)	*E. coli* n (%)	*S. marcescens* n (%)	VRE n (%)
**SDR** **(n = 56)**	Ampicillin-Sulbactam	0 (0.0)	1 (6.7)	0 (0.0)	0 (0.0)	0 (0.0)	1 (50.0)	0 (0.0)
	Amikacin-Gentamicin	1 (5.3)	2 (13.3)	0 (0.0)	0 (0.0)	0 (0.0)	0 (0.0)	0 (0.0)
	Meropenem	9 (47.4)	5 (33.3)	3 (50.0)	1 (16.7)	2 (40.0)	1 (50.0)	1 (100.0)
	Cefoperazone-Sulbactam	0 (0.0)	1 (6.7)	0 (0.0)	0 (0.0)	0 (0.0)	0 (0.0)	0 (0.0)
	Colistin	1 (5.3)	2 (13.3)	1 (16.7)	0 (0.0)	1 (20.0)	0 (0.0)	0 (0.0)
	Cefotaxime	8 (42.1)	4 (26.7)	2 (33.3)	0 (0.0)	2 (40.0)	0 (0.0)	0 (0.0)
	TMP–SMX	0 (0.0)	0 (0.0)	0 (0.0)	5 (83.3)	0 (0.0)	0 (0.0)	0 (0.0)
**MDR** **(n = 10)**	Ampicillin-Sulbactam	2 (18.2)	0 (0.0)	0 (0.0)	0 (0.0)	1 (50.0)		
	Clindamycin	3 (27.3)	0 (0.0)	0 (0.0)	0 (0.0)	1 (50.0)		
	Amikacin-Gentamicin	2 (18.2)	2 (33.3)	0 (0.0)	0 (0.0)	0 (0.0)		
	Meropenem	2 (18.2)	1 (16.7)	1 (50.0)	0 (0.0)	0 (0.0)		
	Cefoperazone-Sulbactam	0 (0.0)	1 (16.7)	0 (0.0)	0 (0.0)	0 (0.0)		
	Piperacillin-Tazobactam	0 (0.0)	1 (16.7)	0 (0.0)	0 (0.0)	0 (0.0)		
	Colistin	1 (9.1)	0 (0.0)	1 (50.0)	0 (0.0)	0 (0.0)		
	Cefotaxime	1 (9.1)	1 (16.7)	0 (0.0)	0 (0.0)	0 (0.0)		

SDR: Single drug resistance; MDR: Multiple drug resistance; TMP–SMX: trimethoprim-sulfamethoxazole; *P. aeruginosa: Pseudomonas aeruginosa*; *K. pneumoniae: Klebsiella pneumoniae*; *A. baumannii: Acinetobacter baumannii*; *S. maltophilia: Stenotrophomonas maltophilia*; *E. coli: Escherichia coli*; *S. marcescens: Serratia marcescens*; VRE: vancomycin-resistant *Enterococcus*. Antibiotic resistance data were analyzed descriptively using multiple response analysis, as individual isolates could exhibit resistance to more than one antibiotic. Therefore, no inferential statistical comparisons were performed between the SDR and MDR groups.

**Table 7:** Firth’s penalized logistic regression model results for patients with MDR.

	OR	95% CI for OR	*p*-Value
Number of comorbidities	1.31	0.35–5.41	0.690
Duration of hospitalization	1.01	0.99–1.03	0.168

## Data Availability

The data presented in this study were obtained from the hospital’s institutional database. Due to ethical and privacy restrictions, the data are not publicly available. Anonymized data may be made available from the corresponding author upon reasonable request and with permission from the relevant institutional authorities.
